# Specific Deletion of AMP-Activated Protein Kinase (α1AMPK) in Murine Oocytes Alters Junctional Protein Expression and Mitochondrial Physiology

**DOI:** 10.1371/journal.pone.0119680

**Published:** 2015-03-13

**Authors:** Michael J. Bertoldo, Edith Guibert, Melanie Faure, Christelle Ramé, Marc Foretz, Benoit Viollet, Joëlle Dupont, Pascal Froment

**Affiliations:** 1 UMR 7247 INRA CNRS Université de Tours Haras Nationaux Physiologie de la Reproduction et des Comportements, 37380, Nouzilly, France; 2 INSERM, U1016, Institut Cochin, Paris, France; 3 CNRS, UMR8104, Paris, France; 4 Université Paris Descartes, Sorbonne Paris Cité, Paris, France; 5 School of Women’s and Children’s Health, Discipline of Obstetrics and Gynaecology, University of New South Wales, Sydney, NSW, Australia; Institute of Zoology, Chinese Academy of Sciences, CHINA

## Abstract

Oogenesis and folliculogenesis are dynamic processes that are regulated by endocrine, paracrine and autocrine signals. These signals are exchanged between the oocyte and the somatic cells of the follicle. Here we analyzed the role of AMP-activated protein kinase (AMPK), an important regulator of cellular energy homeostasis, by using transgenic mice deficient in α1AMPK specifically in the oocyte. We found a decrease of 27% in litter size was observed in ZP3-α1AMPK^-/- ^(ZP3-KO) female mice. Following *in vitro* fertilization, where conditions are stressful for the oocyte and embryo, ZP3-KO oocytes were 68% less likely to pass the 2-cell stage. *In vivo* and in cumulus-oocyte complexes, several proteins involved in junctional communication, such as connexin37 and N-cadherin were down-regulated in the absence of α1AMPK. While the two signalling pathways (PKA and MAPK) involved in the junctional communication between the cumulus/granulosa cells and the oocyte were stimulated in control oocytes, ZP3-KO oocytes exhibited only low phosphorylation of MAPK or CREB proteins. In addition, MII oocytes deficient in α1AMPK had a 3-fold lower ATP concentration, an increase in abnormal mitochondria, and a decrease in cytochrome C and PGC1α levels, suggesting perturbed energy production by mitochondria. The absence of α1AMPK also induced a reduction in histone deacetylase activity, which was associated with an increase in histone H3 acetylation (K9/K14 residues). Together, the results of the present study suggest that absence of AMPK, modifies oocyte quality through energy processes and oocyte/somatic cell communication. The limited effect observed *in vivo* could be partly due to a favourable follicle microenvironment where nutrients, growth factors, and adequate cell interaction were present. Whereas in a challenging environment such as that of *in vitro* culture following IVF, the phenotype is revealed.

## Introduction

Numerous studies have emphasized the importance of adequate nutritional status in maintaining reproductive function. The challenge is to understand the means of communication between the existing nutritional status, energy metabolism and the reproductive system. Ratchford *et al*. have hypothesized that abnormalities in oocyte metabolism, such as that observed in diabetes, could potentially preprogramme the oocyte for poor outcomes after fertilization [1]. Furthermore, Wang *et al*. [2] concluded that maternal diabetes results in various oocyte defects. During oogenesis and folliculogenesis, several dynamic processes that are regulated by endocrine, paracrine and autocrine signals have been shown to be linked with energetic status. For example glucose metabolism is necessary for successful oocyte maturation and the resumption of meiosis [3]. Mitochondria can influence the developmental competence of the oocyte [4]. Indeed, mitochondria play a key role in cellular energy generation, the control of cell death [5] and the dynamic process of meiosis including DNA reorganization [2]. In the case of diabetes, mitochondria are abnormally distributed around the spindle or in the oocyte cytoplasm [2]. For these crucial activities in oocyte maturation, mitochondrial redistribution, activity or dysfunction have been suggested as markers of oocyte quality and are strongly related to fertilization rates and embryo development [2,6].

A protein kinase called AMP-activated protein kinase (AMPK), plays an important role in cellular energy homeostasis and mitochondrial function. AMPK is sensitive to energy content and responds both by stimulating energy production, including glucose and lipid catabolism, and by inhibiting energy-consuming processes such as protein, fatty acid and cholesterol synthesis [7]. AMPK is a heterodimer composed of a catalytic α-subunit bound with β- and γ- regulatory subunits. It is sensitive to the AMP/ATP ratio and is activated by allosteric regulation of increased AMP concentration and by the phosphorylation of the α-subunit at threonine 172 by the upstream kinases; liver kinase B1 (LKB1), Ca^2+^ calmodulin-dependent protein kinase 1 or 2 (CaMKK1 or CaMKK2), TGF-β-activated kinase (TAK1) and possibly kinase suppressor of RAS (KSR2). Apart from its classical functions as a cellular energy sensor, AMPK has a role in the formation and maintenance of cellular junctional complexes and cytoskeleton dynamics [8–10].

In mammals and birds, AMPK has been identified in the different cell types of the ovary (oocyte cumulus, granulosa cells and theca) and in the corpus luteum [11,12]. Its role has been studied in detail in granulosa cell cultures and during oocyte maturation by using pharmacological agents [13–15]. AMPK activators inhibit the secretion of progesterone and / or estradiol by granulosa cells in mammals [12,16]. AMPK improves resumption of oocyte meiosis in mice [13,17–19], whereas pharmacological activation of AMPK blocks nuclear oocyte maturation in pigs and cattle [14,15]. Although AMPK has been shown to be involved in several ovarian functions, no study has yet described the consequences of α1AMPK ablation from the oocyte.

We characterized the role of the α1AMPK subunit in female fertility using an oocyte-specific AMPK knockout model. We have focused on the consequences on cell communication between oocytes and cumulus cells [20], mitochondrial function [21] and early embryo development following *in vitro* fertilization. Improving our understanding of these processes could also be of value in human-assisted reproductive technologies. Indeed, one AMPK activator, called metformin, is currently used as an antidiabetic drug and to treat female infertility associated with insulin resistance.

## Materials and Methods

### Ethics Statement

All animal procedures were carried out in accordance with european legislation for animal experimentation (Directive 86/609/EEC) and with french legislation on animal research. The procedures using oocyte α1AMPK-deficient mice were approved by the ethics committee of Val de Loire (CEEA VdL, Comité d'Ethique pour l'Expérimentation Animale du Val de Loire, n°2012–12–11).

### Animals

Oocyte α1AMPK-deficient mice were obtained by crossing ZP3-Cre mice (C57BL/6-TgN(Zp3-Cre)93Knw, The Jackson Laboratory; Charles River Laboratories, l’arbresle, France) [22] with mice containing floxed α1AMPK subunits on a C57BL/6 background [23,24] in the animal facilities (EU0028, UEPAO, 1297). The ZP3-Cre mice have previously displayed the same fertility characteristics as wild-type mice [22]. Wild-type and mutant mice were maintained under standard conditions of light (12h light, 12h darkness) and temperature with *ad libitum* access to food and water.

The fertility assessment was performed by crossing a 3 month-old wild-type male mouse with two transgenic female mice at the same age for 1 week in the same cage. Two alternative transgenic females were then rotated through a male’s cage. The same male was crossed with the 3 following genotypes of females: 1/ AMPKα1^lox/lox^ mice (control females noted WT, n = 14), 2/ female ZP3-Cre AMPKα1^+/-^ mice (n = 12) and 3/ female ZP3-Cre AMPKα1^-/-^ mice (noted ZP3-KO, n = 20). When a vaginal plug was detected, the mated female was moved in another cage. The litter size was counted at birth and 5 days after birth, to detect neonatal death or cannibalism. Results were presented as number of litters obtained per female at birth, because no neonatal death was observed. We measured 3 litter sizes per female.

To study responsiveness of ovaries to gonadotropins and to recover oocytes and cumulus cell-oocyte complexes (COCs), mice were injected intraperitoneal with 5 IU of equine chorionic gonadotropin (eCG). 46 hours later, 5 IU of human chorionic gonadotropin was administrated (hCG, Intervet, Boxmeer, Holland), A further 12 hours later, oviducal COCs were retrieved in warmed M2 medium (Sigma) or fixed in 4% paraformaldehyde. Animals were killed by cervical dislocation. Ovaries were fixed in bouin solution for histological studies or directly stored at -80°C for biology molecular analysis.

### Oocyte recovery and *In vitro* fertilisation (IVF)

Cumulus oocyte complexes (COCs) were collected from oviducal ampullae in warmed M2 medium (Sigma); then the COCs were washed three times in Embryomax human tubal medium (HTF Medium, Millipore, St Quentin en Yvelines, France). The area of the COC (cumulus expansion) was measured using an image analyser (ImageJ, NIH, USA). Zona pellucida thickness, volume of the cytoplasm and volume of perivitelline space were measured. Four points on the zona were used to calculate the zona thickness per oocyte as described by Jennings *et al*. 2011 [25]. Measurements were performed with 47 ZP3-KO oocytes and 48 WT oocytes retrieved from 6–7 different animals.

COCs were randomly placed into 4-well dishes containing 200 μl of HTF medium per well for *in vitro* fertilization or incubated in hyaluronidase 0.1% for 2 min at 37°C to obtain denuded oocytes. Oocytes were then frozen at -80°C or fixed with 4% paraformaldehyde for 15min.

*In vitro* fertilization (IVF) was performed by recovering fresh sperm from C57BL6 male mice (control mice) from the epididymis into HTF medium. The sperm was placed in the IVF dish containing the mature COCs for 5 hours. After co-incubation, presumptive zygotes were washed three times to remove cumulus cells and excess sperm and placed into 20 μl drops of HTF medium under mineral oil. Embryos were cultured at 37°C in a humidified atmosphere of 5% O_2_ and 6% CO_2_ in air. Embryo assessments were made every 24 h until the 4-cell stage as detailed in Bertoldo *et al*. 2014 [26]. The experiment was repeated 3 times, with a total of at least 80 oocytes inseminated per condition.

### Immunohistochemistry, histological analysis of ovaries and immunofluorescence of oocytes

For immunohistochemistry or immunofluorescence, procedures were described in Tosca *et al*. 2005 and 2006 [11,12]. Sections were incubated overnight at 4°C with 1% Bovine Serum Albumin (BSA)—PBS containing the following primary antibodies at 1:100 dilution: antibody against Cre was purchased from Merck-Novagen (Darmstadt, Germany); phospho-acetyl CoA carboxylase (Ser79) from Upstate Biotechnology Inc., (Lake Placid, NY, USA); α-tubulin, cytochrome C, phosphorylated glycogen synthase kinase 3 beta (phospho-GSK-3β (Ser9)) and Sirt 1 from Cell Signalling (Beverly, MA, USA); β-catenin from Santa Cruz (Santa Cruz, CA, USA); connexin 37 from Alpha Diagnostic (San Antonio TX, USA); N-cadherin and occludin from Sigma (St Louis, MO, USA). Negative controls were rabbit or mouse IgG (Sigma, St Louis, MO, USA). The following day, after two PBS baths for 5 min, sections were incubated for immunofluorescence with secondary antibodies (Alexa fluor 488 goat anti-rabbit IgG or Alexa fluor 488 rabbit anti-mouse IgG (Invitrogen) for 4h at room temperature. Slides were then washed twice for 5 min in PBS and incubated for 10 min with 4’,6’-diamino-2-phenylindole (DAPI; 10 μg/ml, Invitrogen) and mounted with fluorescent mounting medium (Sigma). Immunostaining was performed on ovarian sections from 6 different animals per genotype. Immunofluorescence of denuded oocytes (mitochondria and MII spindle) were performed on at least 42 oocytes retrieved from 6–7 different animals. Mitochondrial staining of live denuded oocytes were realized in M2 medium (Sigma) containing 200 nM Mitotracker ((MitoTracker Orange CM-H2TMRos, M7511, Invitrogen) for 30 min at 37°C. Images were captured using a fluorescent microscope (Zeiss Axioplan 2, Zeiss Gruppe, Jena, Germany) or for oocyte analysis, fluorescent images were captured on a laser scanning confocal microscope (Zeiss Gruppe, Jena, Germany) with a ×63 objective and analysed with ZEN software (Zeiss Gruppe, Jena, Germany). Spindles were analysed according to one- and two-dimensional characteristics in X, Y or Z planes and measurements were calculated regardless of spindle orientation.

### Transmission electron microscopy

Ovaries (n = 4 mice in each genotype) were fixed in 4% glutaraldehyde, 0.1M sodium cacodylate buffer pH 7.4 (Sigma, St Louis, MO, USA) for 24 h at 4°C, post-fixed in 1% osmium tetroxide, and embedded in eponaraldite resin as described in [27]. For ultrastructure analysis, samples were serially sectioned at 70 nm slice thickness, and sections were examined on a CM10 electron microscope (CM 10 Philips, Eindhoven, the Netherlands). Analysis software was used for image acquisition (Soft Imaging System, Olympus, Münster, Germany). Zona pellucida was observed in oocytes of ovarian follicle from 4 different animals per genotype. More than 500 mitochondria per genotype (4 animals/genotype) were analysed and classified as normal or exhibiting altered cristae and considered as abnormal mitochondria. The diameter of mitochondria (μm), distance between the outer and inner mitochondrial membrane (nm), and intracristal space (nm) were measured in at least 85 mitochondria per genotype.

### Western immunoblotting

Oocytes were prepared as detailed in “Oocyte recovery” section, incubated for 2 min at 37°C with 0.1% hyaluronidase to remove cumulus cells, then denuded oocytes were washed two times in PBS and frozen at -80°C. Cumulus cells were retrieved, centrifuged and frozen at -80°C. Lysates were prepared from a group of 40–50 oocytes (or cumulus cells from 40–50 COCs) retrieved from 6–7 different animals. Denuded oocytes and cumulus cells were lysed and exposed to 3 repeated freeze/thaw cycles in lysis buffer. The protein concentration in the supernatant was determined using a calorimetric assay kit (DC assay kit; Uptima Interchim, Montmuçon, France) and equal amounts of proteins were submitted to electrophoresis on SDS-PAGE under reducing conditions and then transferred onto nitrocellulose membranes (Schleicher & Schuell, Ecquevilly, France), as described in Froment *et al*. 2004 [27]. Thereafter, membranes were incubated overnight at 4°C with the following rabbit or mouse antibodies: α1AMPK was purchased from Upstate Biotechnology Inc (Lake, Placid, NY, USA); 3β hydroxysteroid dehydrogenase (3βHSD) and β-catenin from Santa Cruz (Santa Cruz, CA, USA); cytochrome C, extracellular signal-regulated kinase 2 (ERK2), phosphorylated ERK1/2 (pERK1/2), cAMP responsive element-binding protein (CREB), phospho-CREB (Ser 133), histone H3 (H3), peroxisome proliferator-activated receptor-γ coactivator1α (PGC1α) and p53 from Cell Signalling (Beverly, MA, USA); N-cadherin, vinculin from Sigma (St Louis, MO, USA); acetylated H3 (H3 K9/K14) and mdm2 (2A10) from Millipore (Molsheim, France); connexin 37 from Alpha Diagnostic (San Antonio TX, USA). All antibodies were used at 1:1000 dilution in western-blotting, except phospho-CREB antibodies diluted at 1:500. The band densities were quantified using image analysis software (ImageJ, NIH, USA). The results are expressed as the intensity signal in arbitrary units, after normalization by an internal standard (total protein or vinculin) and correspond to the mean of three separate experiments.

### RNA isolation and RT-qPCR

Total RNA was extracted from pools of 50 collected denuded oocytes using NucleoSpin RNA XS (Macherey Nagel, Hoerdt, France) according to the manufacturer’s recommendations. RNA concentrations were measured by spectrophotometry. Following a DNAse treatment (Ambion, Clinisciences, Montrouge, France), 2 μg of total RNA were reverse-transcribed using Super Script II reverse transcriptase (Invitrogen, Cergy Pontoise, France) in the presence of random hexamer primers (Promega, Charbonnieres-les-Bains, France). Real-time quantitative PCRs (Q-PCRs) were performed on Light Cycler 480 (Roche, Meylan, France) as previously described [28]. Briefly, the PCR Master Mix (10 μl) was composed of 4 μl cDNA diluted (5X), 0.2 μl of each primer (200 nM), 2 μl of SsoAdvanced SYBR Green Supermix (Biorad, Marne-la-Coquette, France).

The primer sequences were: forward: 5’ AAACTTGCTAGCGGTCCTCA 3’ and reverse 5’ TGGCTGGTGCCAGTAAGAG 3’ designed and validated for mouse-PGC-1alpha (NM_00894, GenBank, NCBI) [29]; forward: 5’ CCCTAGCAATCGTTCACCTC 3’ and reverse: 5’ TCTGGGTCTCCTAGTATGTCTGG 3’ for mouse-cytochrome b as a marker of a mitochondrial gene (AY999076, GenBank, NCBI) [29]; and forward 5’ AACGACCCCTTCATTGAC 3’ and reverse 5’ GAAGACACCAGTAGACTCCAC 3’ for mouse-glyceraldehyde-3-phosphate dehydrogenase (gapdh) (NM_008084.2, GenBank, NCBI, used as reference gene). 4 pools of 50 ZP3-KO denuded oocytes and 3 pools of 50 wild-type denuded oocytes were analyzed by RT-qPCR. Quantification was performed using Light Cycler integrated software (Roche, Meylan, France) by using the delta-delta Ct method with the use of gapdh as reference gene to normalize expression levels of genes assayed.

### ATP, cAMP and histone deacetylase activity (HDAC) analysis

Four groups of 30–35 denuded oocytes retrieved from 6–7 different animals were lysed by exposing oocytes to 3 repeated freeze/thaw cycles before the commencement of the assays. The protein concentration in the lysate was determined using a calorimetric assay kit (DC assay kit; Uptima Interchim, Montluçon, France). HDAC (NAD^+^-dependent histone deacetylase) activity was assessed using the HDAC-Glo kit according to the manufacturer’s instructions (Promega, Madison, USA). cAMP and ATP concentrations were measured by using the cAMP-Glo Assay (Promega) and the Cell-Titer-Glo Assay (Promega) according to the manufacturer’s instructions. Each standard and sample was assessed in duplicate. The results of each assay were normalized with the protein concentration in each sample.

### Statistical analysis

All data are presented as means ± SEM. A Student’s t test was used to compare means between each genotype. An ANOVA test was performed for QPCR analysis, the means were compared by Fisher’s test. Probability values ≤ 0.05 were considered significant.

## Results

### 1- Reduced fertility in oocyte-specific α1AMPK^-/-^ mutant female mice

**Fig 1:**
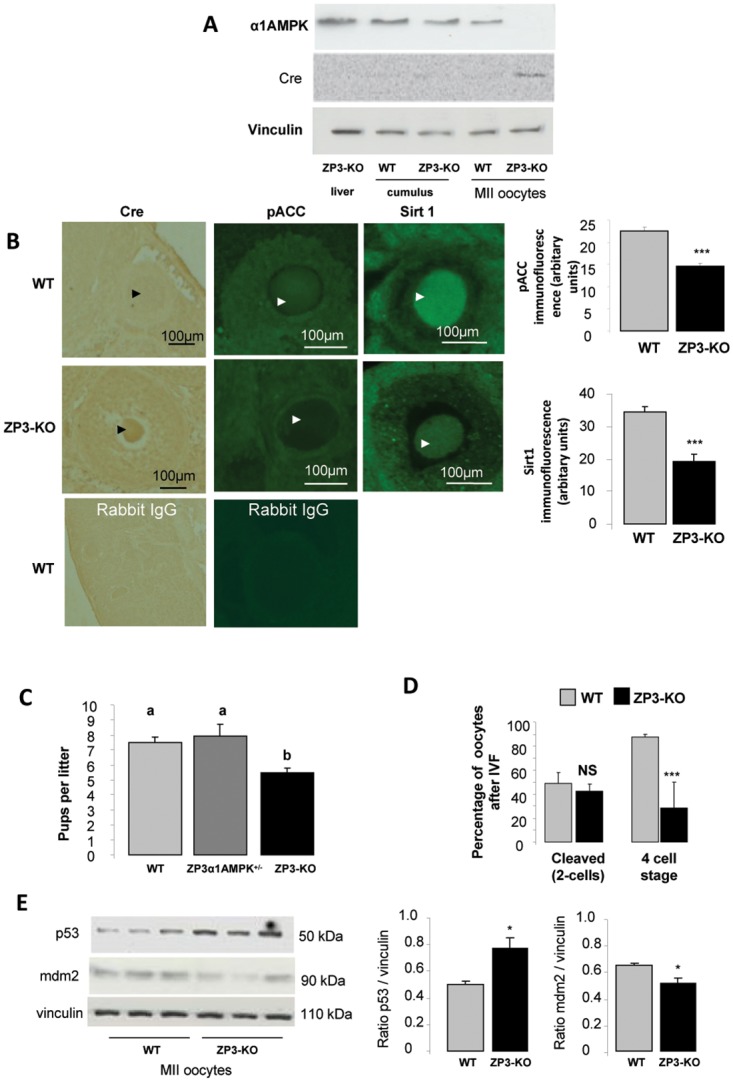
α1AMPK inactivation in oocyte. (**A**) Western blot analysis of the α1AMPK subunit and Cre recombinase in metaphase II (MII) oocytes, cumulus cells and liver retrieved from wild-type and ZP3-KO mice after superovulation (**B**) Conditional invalidation of α1AMPK subunit in the oocyte. Cre expression was localised by immunohistochemistry, and phospho-ACC, and Sirt1, two AMPK substrates, by immunofluorescence in WT and ZP3-KO ovary sections. Arrows show the oocyte (**C**) Fertility analysis of WT (genotype AMPKα1^lox/lox^), heterozygous (genotype ZP3-Cre, AMPKα1^+/-^), and ZP3-KO (genotype ZP3-Cre, AMPKα1^-/-^) female mice were mated with wild-type males. (**D**) Percentage of oocytes cleaved after in vitro fertilization with wild type semen and percentage of cleaved oocytes to reach a 4-cell stage embryo. (**E**) Western blot analysis of p53 and mdm2 protein levels in MII oocytes of WT and ZP3-KO mice. *, P < 0.05 ***, P < 0.001. a, b Values with different letters differ significantly (P < 0.05)

In ZP3-Cre AMPKα1^-/-^ mice (ZP3-KO), Cre recombinase was expressed only in oocytes and was associated with a decrease in phosphorylated Acetyl-CoA carboxylase and Sirt1 expression, two substrates of AMPK (Fig. 1A, B). SIRT1, a nutrient sensor with deacetylase activity, is known to have a reduced downstream signalling in the absence of AMPK [30,31]. Absence of α1AMPK expression was confirmed in metaphase II (MII) oocytes retrieved after superovulation (Fig. 1A, B). Control females (*α1AMPK* ^*loxlox*^, WT), heterozygous (ZP3-Cre AMPKα1^+/-^ mice), and homozygous ZP3-Cre female mice were mated with wild-type males. The mean litter size of ZP3-KO female mice (5.5 ± 0.3 pups/litter) was reduced significantly (*P* < 0.05), by 27%, compared to that of WT or heterozygous female (7.5 ± 0.4 pups/litter) (Fig. 1C). These results were supported by a lower number (*P* < 0.05) of oocytes recovered in ZP3-KO animals (19.1 ± 1.1) compared to WT animals (23.6 ± 1.1) after superovulation.

Following IVF, there were no differences in the ability of oocytes to cleave between ZP3-KO and WT oocytes. However, there was a 68% reduction in the ability of oocyte-specific ZP3-KO embryos to develop to the 4-cell stage (WT mice: 88% vs ZP3-KO: 28%; Fig. 1D). Moreover, in comparison to WT MII oocytes, oocytes recovered from ZP3-KO mice following superovulation presented a higher basal level of p53, a master regulator of cell cycle arrest, associated with a decrease in mdm2, a negative regulator of p53 (Fig. 1E).

**Fig 2:**
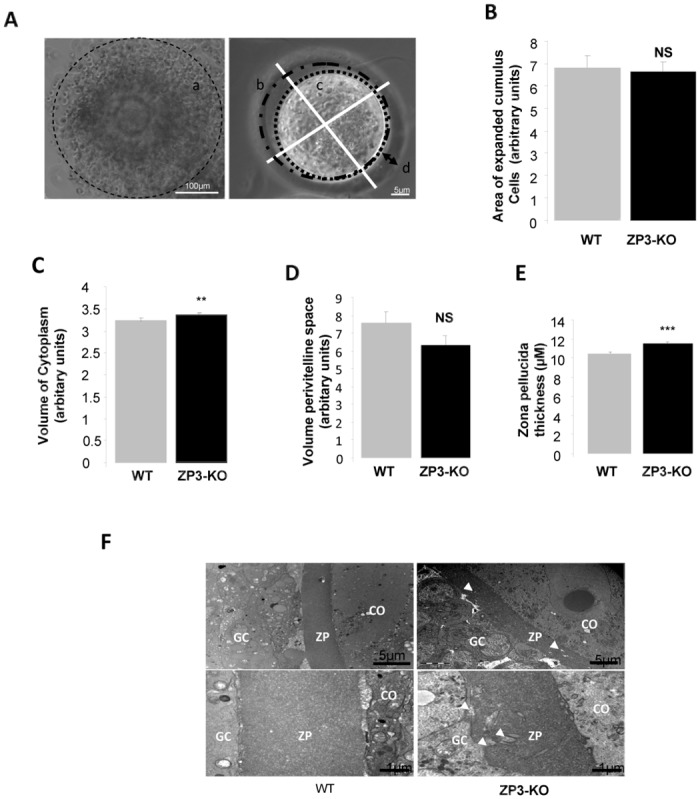
Oocyte morphology. **(A)** Brightfield photomicrographs of the area measured: expanded cumulus 'a'; volume of perivitelline space ‘b’; volume of cytoplasm ‘c’ and zona pellucida thickness ‘d’. Method of measurement of zona pellucida, indicating the points on the zona that were used to calculate the zona thickness per oocyte. **(B)** Analysis of area of cumulus expansion following recovery of COCs 12 h after hCG injection. **(C)** Volume of cytoplasm. **(D)** Volume of perivitelline space. **(E)** Analysis of zona pellucida thickness. **(F)** Transmission electron microscopic micrographs of the zonae pellucidae of WT and ZP3-KO oocytes. ZP: zona pellucida; GC: Granulosa cells; CO: Cytoplasm oocyte. Arrows show invagination in zona pellucidae. **, *P* < 0.01, ***, *P* < 0.001. (n = 40 oocytes/genotypes)

Morphological analysis of retrieved oocytes after superovulation have shown a slight increase in the volume of the cytoplasm (Fig. 2C) and the thickness of the zona pellucida of ZP3-KO oocytes (11.5 ± 0.1 μm) versus WT oocytes (10.4 ± 0.2 μm, *P* < 0.001), but no difference between genotypes in the area of cumulus expansion was observed (Fig. 2A-E). Electron microscopic observations have shown a uniform appearance of the zona pellucida in WT oocytes *in vivo*, but a lack of uniformity of the zona pellucida in ZP3-KO oocytes (Fig. 2F).

### 2- Absence of α1AMPK perturbs cell-to-cell communication

**Fig 3:**
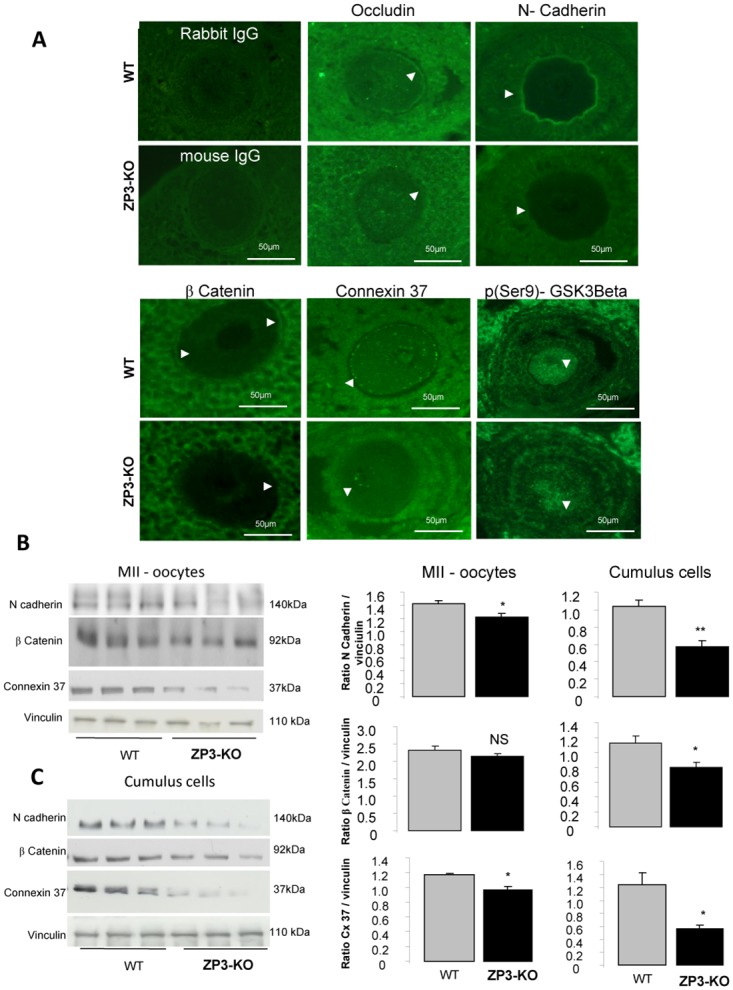
Oocyte cell-to-cell communication. **(A)** Immunofluorescent expression and localization of occludin (a marker for tight junctions), N-cadherin and β-catenin (markers for adherens junctions), connexin 37 (a marker for gap junctions) and phosphorylated GSK3β (for β-catenin signalling) in germinal vesicle stage in WT and ZP3-KO oocytes. **(B and C)** Western blot analysis of N-cadherin, β-catenin and connexin 37 in Metaphase II oocytes and cumulus cells of WT and ZP3-KO mice. Quantification of proteins in both genotypes is shown on the side of the western-blot. *, *P* < 0.05, **, *P* < 0.01.

Junctions were observed between oocytes and the cumulus cells of the ovarian follicle. These junctions are known to support nutrient transfer between the germ and somatic compartments and to allow the transfer of molecules involved in oocyte maturation from the granulosa/cumulus cells to the oocyte [32]. We localized junctional proteins between oocytes and their surrounding cumulus cells. *In vivo*, in ZP3-KO oocytes, expression of occludin, a component of tight junctions, was less common at the interface and more localized in the cytoplasm of oocytes (Fig. 3A). β-catenin and N-cadherin, two components of adherens junctions were localized at the oolemma interface of the oocyte and in the zona pellucida/cumulus interface. They were reduced in ZP3-KO mice (Fig. 3A, B). The glycogen synthase kinase-3β (GSK3β), reported to mediate β-catenin/Wnt signalling [33,34], also showed a reduced intensity of labelling in the cytoplasm of ZP3-KO oocytes (Fig. 3A). In addition, the localization of connexin37, a critical gap junction protein for oocyte/cumulus cell communication, was barely detectable in ZP3-KO oocytes at the zona pellucida, in contrast to wild-type oocytes (Fig. 3A, B). A similar reduction in N-cadherin, β-catenin and Connexin37 protein expression in isolated cumulus cells purified from superovulated ZP3-KO COCs was observed (Fig. 3C).

**Fig 4:**
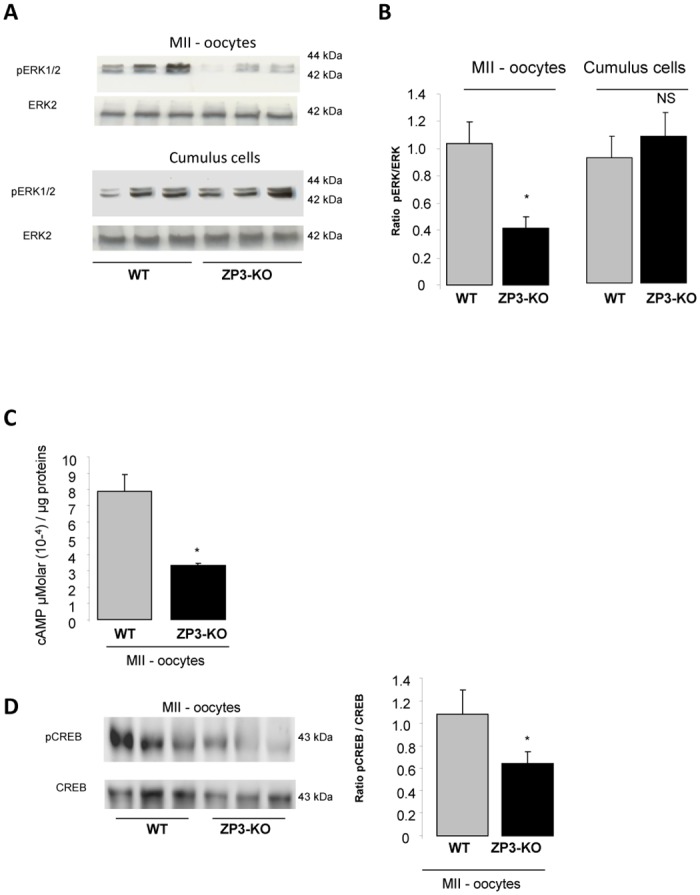
ERK and cAMP content in oocyte. **(A)** Western blot analysis of phosphorylated ERK1/2 expression in MII oocytes and cumulus cells of WT and ZP3-KO mice and **(B)** quantification of proteins in both genotypes is shown on the side of the western-blot. **(C)** Concentration of cAMP in MII WT and ZP3-KO oocytes. **(D)** Western blot analysis of phospho-CREB expression in MII oocytes of WT and ZP3-KO mice. Total CREB served as a loading control. Quantification of proteins is shown on the side of the western-blot. Results are representative of 3 independent experiments. *, *P* < 0.05.

Both PKA and MAPK signal transduction pathways were observed to be involved in the junctional communication between the cumulus/granulosa cells and the oocyte and the somatic cells of the follicle. Communication is essential for the growing oocyte and has a role in supplying nutrients to the maturing oocyte [35]. Western-blot analyses have revealed a significantly reduced phosphorylation of a MAP protein, the extracellular signal-regulated kinases (ERK1/2) in ZP3-KO MII oocytes when compared with wild-type oocytes (Fig. 4A, B), but not in the ZP3-KO cumulus cells (Fig. 4A, B). Concomitantly, we also observed a reduction in the protein kinase A (PKA) activity as revealed by the diminution of CREB phosphorylation (a target of PKA) and cAMP content (cAMP activates PKA) in ZP3-KO MII oocytes (Fig. 4C, D).

### 3- Energy production is impaired in the absence of α1AMPK in oocyte

**Fig 5:**
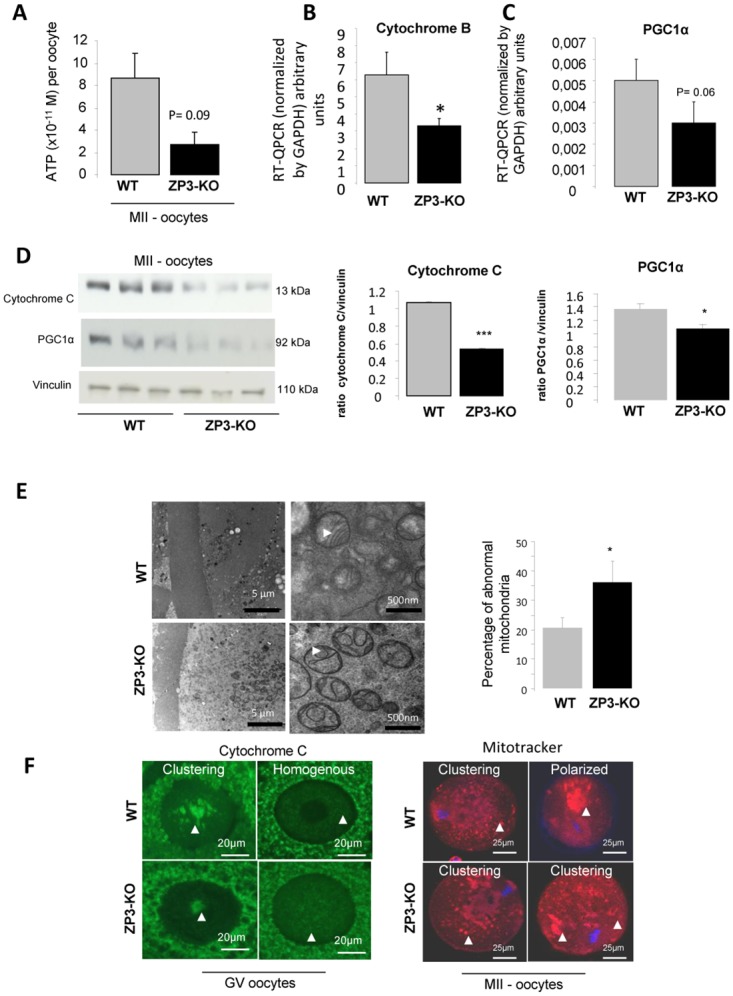
Energy production is impaired in the absence of α1AMPK in oocyte. **(A)** Concentration of ATP in a MII oocyte of WT and ZP3-KO mice. **(B, C)** Analysis of mitochondrial gene expression by RT-QPCR (cytochrome B and PGC1α, normalized by cyclophilin expression) and **(D)** by western blot for cytochrome C and PGC1α protein in WT and ZP3-KO oocytes at metaphase II stage. **(E)** Mitochondrial ultrastructure GV oocytes analysed by transmission electron microscopy. Arrows show crest, intermembrane space and vacuolization in mitochondria. Abnormal mitochondria were quantified. **(F)** Mitochondrial distribution pattern in GV and MII oocytes. Distribution pattern of mitochondria (see arrow) localized in GV oocytes using immunofluorecence against cytochrome C in WT and ZP3-KO follicle. Two patterns of distribution were observed; clustering and homogeneous. Mitochondrial distribution pattern (arrow) in MII oocytes was visualized after fluorescent Mitotracker labelling. DNA was counterstained with DAPI. Clustering and polarized patterns of distribution were observed in MII WT oocytes whereas only a clustering pattern of distribution was observed in ZP3-KO oocytes *, *P* < 0.05, ***, *P* < 0.001.

When mutant oocytes were placed into a stressful environment, such as *in vitro* culture, a notable reduction in fertility was observed. This suggested that in the absence of α1AMPK, energy production within the oocyte was perturbed. Thus, we measured the ATP content in ZP3-KO oocytes, which was about 3-fold lower than in WT oocytes (Fig. 5A). Expression of mitochondrial genes showed a decrease in cytochrome B and PGCα1 at the transcript levels (Fig. 5B, C) and a decrease in cytochrome C and PGCα1 at the protein level in ZP3-KO oocytes compared to control oocytes (Fig. 5D). Furthermore when mitochondria were analysed by transmission electron microscopy, ZP3-KO oocytes presented with altered morphology. Mitochondria displayed a poorly structured matrix, numerous vacuoles, a narrow inter membrane space and rupture of the outer membrane (Fig. 5E). Indeed, in ZP3-KO oocytes, there was a greater proportion (38.1 ± 7.0%) of mitochondria with altered cristae when compared with WT mitochondria (20.6 ± 3.4%; *P* < 0.05) (Fig. 5E). In addition, characteristics associated with swollen mitochondria [36] were observed in ZP3-KO oocytes when compared with mitochondria from WT oocytes. These included a larger mitochondrial diameter and distance between the outer and inner mitochondrial membrane (Table 1). No difference was observed between genotypes in the intracristal space of mitochondria.

**Table 1:** Analysis of mitochondrial ultrastructure.

Genotype	percentage of mitochondria with altered cristae	diameter mitochondria (μm)	distance between the outer and inner mitochondrial membrane (nm)	intracristal space (nm)
WT	20.6 ± 3.4	0.55 ± 0.02	25.6 ± 1.8	31.8 ± 1.7
ZP3-KO	36.2 ± 7.0	0.67 ± 0.01	31.1 ± 1.5	35.3 ± 1.8
p =	0.02	P<0.0001	0.02	0.22
	*	***	*	Ns

data are presented as means ± SEM.

*, *P* < 0.05;

***, *P* < 0.001;

NS: non significant.

In the growing follicle, two mitochondrial distributions were observed in both genotypes at the germinal vesicle (GV) stage: 1/ the apparent clustering of mitochondria around the GV (Fig. 5F), as reported previously [2]; and 2/ a uniform distribution where the mitochondria were dispersed throughout the cytoplasm. However, with the completion of meiotic maturation, we observed clustering and polarized patterns of mitochondrial distribution in WT oocytes at the MII stage (Fig. 5F), regarded as a normal distribution pattern [2]. In contrast, ZP3-KO oocytes presented only a clustering pattern of distribution and no polarized distribution (Fig. 5F).

### 4- α1AMPK is not required for spindle formation but reduces HDAC activity

**Fig 6:**
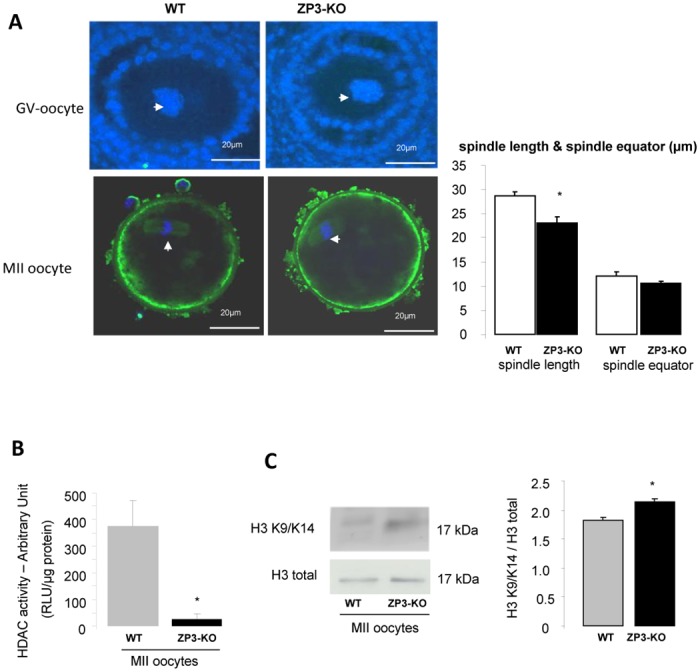
α1AMPK is not required for spindle formation but reduces HDAC activity. **(A)** Configuration of DNA in GV oocytes and in ovulated MII oocytes in WT and ZP3-KO mice was analysed on confocal microscope after immunofluorescence. Spindle (green) was stained with α-tubulin antibody and DNA was counter-stained with DAPI (see arrow). Morphometric parameters were measured for each spindle: spindle length and spindle equator (μm). Scale bar: 20 μm. **(B)** Analysis of HDAC activity in MII WT and ZP3-KO oocytes. **(C)** Western blot analysis of acetylated histone H3 at lysine 9 and 14. Quantification of proteins in both genotypes is shown on the side of the western-blot. *, *P* < 0.05.

Because pharmacological activators of AMPK lead to resumption or blockage of oocyte meiosis [13,17–19], we analysed spindle formation in MII oocytes. Analysis using confocal microscopy did not detect differences in nuclear morphology at GV stage or in MII oocytes between wild-type or mutant animals. (Fig. 6A). However when spindle length was measured, we observed shorter spindles in ZP3-KO (23.1 ± 1.4 μm) oocytes when compared to WT oocyte spindles (28.8 ± 0.9 μm; *P* < 0.05). There were no differences between the size of the spindle equator and chromosomes were well-aligned on the metaphase plate from both genotypes. However because AMPK is linked to the deacetylase Sirt, we measured the level of histone deacetylase (HDAC) activity. We observed a decrease in HDAC activity in ZP3-KO MII oocytes (Fig. 6B), and a slight increase in histone H3 acetylation at lysine 9 (H3K9) and lysine 14 (H3K14) residues (Fig. 6C).

## Discussion

In the present study, we observed that the absence of α1AMPK can directly affect the oocyte and its maternally-derived mitochondria. We also demonstrated that the expression of key proteins involved in oocyte/cumulus cell communication, such as connexin 37, is reduced and signalling pathways associated with cell communication are altered (MAPK and PKA) in the absence of α1AMPK. Moreover, chromosome morphology at the MII stage did not seem to be modified. However spindle length is reduced and an increase in acetylation is associated with the knock-out of the α1AMPK gene. The harmful consequences of absence of α1AMPK were stronger in oocytes analysed *in vitro* than *in vivo*. Together, these results could explain the reduction in fertility that was observed in oocyte ZP3-KO animals. However, we cannot exclude the possibility that some of the observed effects are due to the Cre expression in the oocyte.

Communication between cumulus cells and oocytes is crucial for oocyte meiotic maturation and to acquire full developmental competence. Communication between the oocyte and the surrounding cumulus cells is established by the opening of bidirectional channels. We have localized proteins involved in junction communication between the germinal and somatic compartments (connexin37, N-cadherin, β-catenin and occludin) [37–39]. A reduction in connexin37 between the zona pelucida in ZP3-KO MII oocytes and their cumulus cells suggests a probable reduction in gap junction communication. Connexin37 is known to be a critical gap junction protein in oocytes, as described in the knock-out mouse model for connexin37 [40,41]. The results in ZP3-KO oocytes are not unusual, because AMPK has already been reported to regulate ion channels. For example, AMPK down-regulates connexin26 in *Xenopus* oocytes [42] and a potential role for AMPK in the activity of hemichannels was described recently [43]. Furthermore in a diabetic mouse model, where oocyte quality is poor, there is decreased connexin26 and connexin37 expression and oocyte-somatic gap junction communication [44]. In our model, as in connexin37^-/-^ mice, electron microscopic analysis of oocytes has shown that junctions between granulosa and oocytes were altered or absent [41]. In addition to gap junctions, oocytes interact with granulosa cells through adhesion junctions composed of protein such as E-cadherin and N-cadherin [38,45]. Expression of N-cadherin increases throughout maturation, fertilization and early embryogenesis [46]. Thus, N-cadherin mediated cell contact is associated with the maintenance of meiotic arrest [37]. The reduction of N-cadherin early in ZP3-KO mice raises the possibility of a defect during oocyte maturation.

During recent decades, several groups have associated the MAPK and PKA pathways in the regulation of proteins involved in junction communication. Thus, expression of connexin43 in cumulus cells was down-regulated after the LH surge. The use of a treatment with a specific inhibitor of MAPK kinase inhibits the connexin43 down-regulation [47,48]. Moreover, activation of MAPK in oocytes is important for oocyte maturation induced by FSH and is more closely associated with post-germinal vesicle breakdown events such as meiotic spindle organization [49,50]. In addition, N-cadherin binding could stimulate intra-oocyte cAMP through the GPR3 receptor [51]. High cAMP concentrations in cumulus cells, oocytes, or both lead to a prolonged oocyte-cumulus cell communication and delayed meiotic resumption [52,53]. Indeed, it is well known that a decrease in intra-oocyte cAMP is important for meiosis and for acquisition of oocyte competence in numerous species [54–56]. These results suggest that in ZP3-KO oocytes, the alteration of cell communication between the oocyte and cumulus has repercussions on the MAPK and PKA pathway and potentially in the acquisition of developmental competence.

As the consequences of ablation of α1AMPK from oocytes cultured *in vitro* are stronger than *in vivo* (the low percentage of 4-cell embryos cultured *in vitro* versus small reduction in pup number), we supposed that nutrient availability and energy production were important. Indeed, it is also known that oocytes and embryos in a hyperglycaemic environment have reduced developmental competence [57–59]. We have focused on mitochondrial activity, which is responsible for ATP synthesis and energy accumulation during oogenesis. This function is critical for embryo developmental competence [60]. In the present study we observed a lower concentration of ATP in ZP3-KO oocytes associated with an overall reduction in the quantity and quality of mitochondria, similar to that previously observed in *α1AMPK* ^*-/-*^ male germ cells [61]. We note that PGC1α, a master regulator of mitochondrial biogenesis [62,63] and known to be up-regulated by AMPK and MAPK, was down-regulated in our oocyte model. A study using a type I diabetic mouse model has reported similar consequences at the mitochondrial level in oocytes. For example, although the pattern of localization of mitochondria in ZP3-KO oocytes appeared to be normal for immature stage oocytes as reported by Wang *et al*. [2], the mature stage MII oocytes of ZP3-KO mice present a clustering pattern of mitochondria suggesting an altered mitochondrial localization as in diabetic mice [2]. Moreover, oocytes from diabetic mice and ZP3-KO mice show an increase in abnormal mitochondria. Similarly to ZP3-KO, diabetic oocytes have also shown low activation of AMPK and its target proteins such as acetyl-CoA carboxylase [1,2,44]. These results are in agreement with Egan *et al*. [64] where in mouse embryonic fibroblasts, loss of AMPK or ULK1 (an AMPK substrate) resulted in abnormal mitochondria.

While a 27% reduction in litter size was observed in ZP3-KO mice following natural mating, we observed an increase in the number of mutant zygotes arresting at the 2-cell stage following IVF, suggesting that mutant embryos were not adapted to the stresses experienced during culture. It is likely that the normal processes of oocyte maturation were not faithfully completed [65] in an environment where nutrient regulation is not optimal, suggesting that activation of metabolic sensors seems to be crucial for embryo development in stressful conditions. A previous report has reinforced this hypothesis, because the quality of oocytes from diabetic mice, which are metabolically perturbed, can be restored using the AMPK activator AICAR [1]. In addition, under hyperglycaemic conditions, the use of metformin lead to AMPK and SIRT1 activation and was associated with a decrease in proapoptotic p53 protein abundance [66–68]. In our study, absence of α1AMPK in the oocyte decreased mdm2 protein level, a strong negative regulator of p53, leading to an increase in the p53 content and probably inducing a cell cycle arrest. However, further investigations are needed to learn the exact role of AMPK under stress conditions (i.e. *in vitro* conditions or under-nutrition before fertilisation and during embryo development).

Consequences for oocyte maturation before fertilization were investigated by showing the spindle formation in metaphase II. Indeed, recently it has been shown that AMPK colocalised with γ-tubulin during metaphase I and II stages, suggesting that AMPK could have a role in spindle function [69]. However, treatment of oocytes with AMPK inhibitor (compound C) did not prevent spindle formation or migration, as observed in the α1AMPK^-/-^ oocyte. An hypothesis is that other AMPK related kinases (Salt-inducible Kinase: SIK1, SIK2; Microtubule-Associated protein-Regulating Kinase, MARK) present in the gonad could be allowed to compensate for this function in the absence of AMPK. Although we saw a decrease in spindle length, the absence of α1AMPK does not appear to be necessary for proper spindle function or gross morphology. However, α1AMPK could be involved in chromatin remodelling, because we observed a slight increase in acetylation of H3 histone in oocytes from ZP3-KO mice. These data are correlated with reduction in HDAC activity, and Sirt1 expression *in vivo*, a histone deacetylase protein regulated by α1AMPK. Overall, it suggests that AMPK can modify oocyte proteins and histone acetylation status. These observations could be linked to other reports such as those relating to the aorta and heart tissue where a decrease in AMPK and SIRT1 expression is associated with an increased H3 acetylation [70]. Interestingly, acetylation of histones H3 and H4 appear to be linked to an overexpression of connexin43 in a prostate cell line [71,72] and PGC1α and p53 can change their availability [66–68]. Moreover, the inadequate histone deacetylation causes changes in gene expression, which can lead to embryopathy in mice [73].

Finally, while we observed gross abnormalities in mitochondria and junctional proteins, we found only a moderate failure of fertility in mutant animals in comparison with the development of embryos *in vitro*. But AMPK is also known to be a kinase sensitive to different stresses such as energy depletion and temperature change [74]. It has been shown that under metabolic, oxidative and osmotic stresses *in vitro*, AMPK is required for normal nuclear maturation [75]. Therefore it could be envisaged that in the absence of AMPK under stressful conditions *in vitro*, the ability to limit the harmful consequences was severely compromised and resulted in embryo arrest. The relatively non-stressful *in vivo* environment would explain the discrepancies between the number of pups per litter and *in vitro* embryo development.

In conclusion, this is the first study to describe the absence of α1AMPK specifically in the oocyte and its effect on fertility. It shows that absence of AMPK, which is a key energy sensor, altered oocyte mitochondrial function, the bidirectional communication between the oocyte and its adjoining cumulus cells, and acetylation status. Together these results suggest a reduction of oocyte developmental competence and raise questions about the oocyte-specific action of AMPK in cases of metabolic disorders such as insulin resistance and polycystic ovary syndrome.
